# Functional Genomic Analyses of the 21q22.3 Locus Identifying Functional Variants and Candidate Gene *YBEY* for Breast Cancer Risk

**DOI:** 10.3390/cancers13092037

**Published:** 2021-04-23

**Authors:** Chris Shidal, Xiang Shu, Jie Wu, Jifeng Wang, Shuya Huang, Jirong Long, Joshua A. Bauer, Jie Ping, Xingyi Guo, Wei Zheng, Xiao-Ou Shu, Qiuyin Cai

**Affiliations:** 1 Vanderbilt Epidemiology Center, Vanderbilt-Ingram Cancer Center, Department of Medicine, Division of Epidemiology, Vanderbilt University School of Medicine, Nashville, TN 37203, USA; chris.shidal@vumc.org (C.S.); ShuX@mskcc.org (X.S.); jie.wu@vumc.org (J.W.); xywang0426@126.com (J.W.); huangsya@sdu.edu.cn (S.H.); jirong.long@vumc.org (J.L.); Jie.ping.1@vumc.org (J.P.); xingyi.guo@vumc.org (X.G.); wei.zheng@vumc.org (W.Z.); xiao-ou.shu@vumc.org (X.-O.S.); 2 Memorial Sloan Kettering Cancer Center, Department of Epidemiology & Biostatistics, New York, NY 10075, USA; 3 Department of Breast Surgery, The Second Hospital, Cheeloo College of Medicine, Shandong University, Jinan 250033, China; 4 Department of Biochemistry, Vanderbilt University School of Medicine, Nashville, TN 37203, USA; joshua.a.bauer@vanderbilt.edu

**Keywords:** breast cancer, YBEY gene, functional assays

## Abstract

**Simple Summary:**

Previous research has revealed a genetic predisposition to breast carcinogenesis. Thus, identifying causal genetic variants and their associated gene networks may improve breast cancer diagnostics and risk assessment. Our study investigated *YBEY*, an uncharacterized gene in humans, and its functions in breast cancer risk and progression. We identified two genetic variants associated with *YBEY* expression that may have causal functions in breast cancer risk. We performed in vitro functional assays using MCF-7, T47D, and MDA-MB-231 breast cancer cell lines and showed that knockdown of *YBEY* expression significantly inhibited proliferation, colony formation, and invasion/migration. We utilized RNA sequencing to identify gene networks associated with *YBEY* knockdown including inflammation and metabolic pathways. Further, we used data available in The Cancer Genome Atlas to explore trends in *YBEY* expression patterns in normal and tumor tissues. Our study provides a role for *YBEY* in breast carcinogenesis, and further studies investigating its mechanistic functions are warranted.

**Abstract:**

We previously identified a locus at 21q22.3, tagged by the single nucleotide polymorphism (SNP) rs35418111, being associated with breast cancer risk at a genome-wide significance level; however, the underlying causal functional variants and gene(s) responsible for this association are unknown. We performed functional genomic analyses to identify potential functional variants and target genes that may mediate this association. Functional annotation for SNPs in high linkage disequilibrium (LD, r^2^ > 0.8) with rs35418111 in Asians showed evidence of promoter and/or enhancer activities, including rs35418111, rs2078203, rs8134832, rs57385578, and rs8126917. These five variants were assessed for interactions with nuclear proteins by electrophoretic mobility shift assays. Our results showed that the risk alleles for rs2078203 and rs35418111 altered DNA-protein interaction patterns. Cis-expression quantitative loci (cis-eQTL) analysis, using data from the Genotype-Tissue Expression database (GTEx) European-ancestry female normal breast tissue, indicated that the risk allele of rs35418111 was associated with a decreased expression of the *YBEY* gene, a relatively uncharacterized endoribonuclease in humans. We investigated the biological effects of *YBEY* on breast cancer cell lines by transient knock-down of *YBEY* expression in MCF-7, T47D, and MDA-MB-231 cell lines. Knockdown of *YBEY* mRNA in breast cancer cell lines consistently decreased cell proliferation, colony formation, and migration/invasion, regardless of estrogen receptor status. We performed RNA sequencing in MDA-MB-231 cells transfected with siRNA targeting *YBEY* and subsequent gene set enrichment analysis to identify gene networks associated with *YBEY* knockdown. These data indicated *YBEY* was involved in networks associated with inflammation and metabolism. Finally, we showed trends in *YBEY* expression patterns in breast tissues from The Cancer Genome Atlas (TCGA); early-stage breast cancers had elevated *YBEY* expression compared with normal tissue, but significantly decreased expression in late-stage disease. Our study provides evidence of a significant role for the human *YBEY* gene in breast cancer pathogenesis and the association between the rs35418111/21q22.3 locus and breast cancer risk, which may be mediated through functional SNPs, rs35418111 and rs2078203, that regulate expression of *YBEY*.

## 1. Introduction

The Surveillance, Epidemiology, and End Results program estimates that approximately 276,000 new breast cancer cases will be diagnosed within the United States in 2020, accounting for over 15% of all newly diagnosed cancer cases [1]. While improved diagnostics and treatments for breast cancer have significantly enhanced patient prognosis in recent years, the need to understand the underlying molecular mechanisms of breast carcinogenesis is the focus of much research. Genetic factors driving breast carcinogenesis have been revealed to have profound impacts on risk assessment, prognosis, and treatment. Hereditary pathogenetic mutations in known breast cancer susceptibility genes (e.g., *BRCA1*) only explain a small percentage (5–10%) of breast cancer cases in the general population [2]; thus, identifying genetic variants and their associated gene networks that contribute to breast cancer risk remains paramount. Further studies investigating genetic regulatory mechanisms in breast cancer etiology will expand breast cancer risk assessment and potential intervention strategies.

Our group recently identified genetic variant rs35418111 to be associated with breast cancer risk in a genome-wide association study (GWAS) [3]. Single nucleotide polymorphism (SNP), rs35418111, is located in the intronic region of the pericentrin (*PCNT*) gene. Expression quantitative trait loci (eQTL) analysis indicated that rs35418111 is associated with the expression of the uncharacterized human *YBEY* (*C21orf57*) gene, which is proximal to *PCNT* [3]. While *YBEY* has no known function in humans, the bacterial *Ybey* protein functions as a metalloendoribonuclease, with explicit roles in ribosomal RNA (rRNA) biogenesis and maturation [4,5]. Studies in bacteria and human cell lines demonstrate that *YBEY* localizes in the mitochondria and is an essential gene for cell growth and mitochondrial function [6,7]. Further, *Ybey* has been shown to regulate chloroplast processing in plants [8], establishing the conserved nature of this gene. A prior exome sequencing study conducted in a Chinese population identified rs13047478 to be associated with breast cancer risk, and subsequent eQTL analysis indicated that this variant was associated with *YBEY* expression [9]. Additionally, *YBEY* has been suggested as a potential causal gene for colorectal adenomatous polyposis [10]. This evidence suggests that human *YBEY* may play a significant role in cancer pathogenesis.

In our study, we explored the biological relevance of the *YBEY* gene in human breast cancer cell lines using in vitro functional assays to assess proliferation, colony formation, and invasion/migration. We also performed RNA sequencing (RNA-seq) following siRNA-mediated knockdown (k.d.) of *YBEY* mRNA to identify genes and gene networks significantly associated with human *YBEY*. We also performed electrophoretic mobility shift assays (EMSA) to identify potential causal variants which were in high linkage disequilibrium (LD) with the index SNP, rs35418111. Our results show, for the first time, that several genetic variants may play a role in breast cancer risk through modulation of human *YBEY* expression.

## 2. Materials and Methods

### 2.1. Functional Annotation

Following our previous work [11], we identified putative functional SNPs in strong LD (r^2^ > 0.8 in Asian populations) for the GWAS-identified SNP, rs35418111, using 1000 Genomes project data from HaploReg v4 [12] (Broad Institute of MIT and Harvard, Cambridge, MA, USA). The putative functional SNPs, with evidence of promoter or enhancer activities, annotated either from The Encyclopedia of DNA Elements (ENCODE, National Institutes of Health, Bethesda, MD, USA) and/or the Roadmap Epigenomics Mapping Consortium (ROADMAP) projects (National Institutes of Health), were selected for further analysis. We prioritized variants for in vitro functional assays based on their evidence of breast cancer-related transcription factor DNA-bindings and motifs, chromatin accessibility sites, and histone modifications.

### 2.2. Cis-eQTL

We extracted cis-eQTL results for the GWAS-identified SNP, rs35418111, and nearby genes (within 1Mb to the lead SNP) from the Genotype-Tissue Expression v8 (GTEx, Broad Institute of MIT and Harvard) database, based on normal breast tissues (detailed analysis in [3]). In addition, target genes from HaploReg v4.1 were also evaluated based on the above functional annotation.

### 2.3. Cell Culture

The human breast cancer cell lines, MDA-MB-231 and MCF-7, were obtained from the American Type Culture Collection (ATCC, Manassas, VA, USA). The T47D cell line was a kind gift from Jennifer Pietenpol (Vanderbilt-Ingram Cancer Center, Nashville, TN 37203, USA). MDA-MB-231 is a triple-negative breast cancer cell line, while MCF-7 and T47D lines are estrogen receptor (ER) positive lines. All three cell lines were cultured in Dulbecco’s Modified Eagle Medium (DMEM, Thermo Fisher, Waltham, MA, USA) supplemented with 10% fetal bovine serum (FBS, Thermo Fisher) and 1% penicillin-streptomycin (Thermo Fisher) at 37 °C with 5% CO_2_ in a humidified incubator. All cell lines were monitored for mycoplasma contamination using the colorimetric MycoAlert detection kit (Lonza, Basel, Switzerland) throughout the course of the study. All experiments were performed on cells within a passage number less than ten, following an initial passage of the cryopreserved cells.

### 2.4. Electrophoretic Mobility Shift Assays (EMSA)

SNPs to be assessed by EMSA were selected using the HaploReg v4.1 database and were in high LD with rs35418111. SNPs were prioritized based on a combination of eQTL hits, number of motif changes, and allele frequency in Asian and European populations. Nuclear lysates from each cell line were obtained using the NE-PER Nuclear and Cytoplasmic Extraction kits (Thermo Fisher). Briefly, 1 × 10^6^ breast cancer cells per well were seeded in 6-well culture treated plates for 48 h and subsequently harvested by trypsinization. Cells were washed twice with phosphate buffered saline (PBS, Thermo Fisher), pelleted by centrifugation at 1000 rpm for 10 min, and protein lysates were extracted following the manufacturer’s recommended protocols. EMSAs were performed using the LightShift Chemiluminescent EMSA kit (Thermo Fisher) following the manufacturers recommended protocols. Optimization of DNA-protein binding was performed by varying the final concentrations of magnesium chloride in the sample. DNA-protein complexes were run on a 6% polyacrylamide gel at 100 V for 60 min. Complexes were transferred to a nylon membrane at 100 V for 30 min and crosslinked using a UV Stratalinker 2400 UV Crosslinker (Stratagene, Santa Clara, CA, USA). Chemiluminescent detection was performed using the Amersham Imager 600 (GE Healthcare, Chicago, IL, USA). Sequences for the reference and alternate oligos for the alleles assayed are provided in Table S1. Full gel images are provided in Figure S1.

### 2.5. RNA Interference

MDA-MB-231, T47D, and MCF-7 cells were plated at 1.25 × 10^5^ cells/well in 6-well plates and reverse-transfected using three siRNAs (Y1, Y2, and Y3) targeting different sites of *YBEY* mRNA (Dharmacon, Lafayette, CO, USA) at 10 nM using liposomal delivery (RNAiMAX, Life Technologies, Carlsbad, CA, USA). The sequences for each siRNA have been provided in Table S2. A non-targeting control (NTC) siRNA (AllStars Neg Control siRNA, Qiagen, Germantown, MD, USA) and a positive control (POS) siRNA (AllStars Hs Cell Death Control siRNA, Qiagen) were used as the negative control and the positive control, respectively. The k.d. efficiency was assessed after 24 h and up to 48 h post-transfection by quantitative real-time PCR (qPCR) using glyceraldehyde-3-phosphate dehydrogenase (GAPDH) as a housekeeping gene. These data represent fold-changes normalized to the negative control and have been provided in Figure S2.

### 2.6. RNA Isolation and qPCR

Following transfection, RNA was isolated from breast cancer cells using the miRNeasy Mini Kit (Qiagen) according to the manufacturer’s protocol. cDNA was synthesized by using the High-Capacity cDNA Reverse Transcription Kit (Thermo Fisher). Amplification and quantitation were performed on an Applied Biosystems 7900HT Fast Real-Time PCR system (Thermo Fisher) equipped with Relative Quantitation (RQ) Manager software using the Luna Universal qPCR Master Mix (New England BioLabs, Ipswich, MA, USA). Relative mRNA expression levels for each gene and condition were calculated using the ∆∆Ct method. GAPDH was used as the reference gene. Sequences for each primer have been provided in Table S3.

### 2.7. Cell Viability

Cell viability was assessed using the alamarBlue™ Cell Viability Reagent (Thermo Fisher). MDA-MB-231, T47D, and MCF-7 cells were plated at 5 × 10^3^ cells/well in 100 µL of DMEM in cell culture treated 96-well plates (Corning Costar, Corning, NY, USA) and transfected with siRNAs targeting *YBEY* mRNA. 96 h post-transfection, 10 µL of alamarBlue reagent was added to each well. Plates were then incubated at 37 °C for 2–4 h, and subsequently, fluorescence was measured on a BioTek Synergy HT plate reader (BioTek, Winooski, VT, USA) with excitation at 570 nm and emission at 585 nm. Relative fluorescence for each condition was averaged and normalized to the negative control and presented as percent of control. Results represent data obtained from three independent experiments performed in quadruplicate.

### 2.8. Colony Formation Assay

After 16 h post-transfection, the siRNA-transfected cells were harvested and re-seeded in 6-well plates at a density of 2000 cells per well in 2 mL of antibiotic-free culture media and allowed to proliferate for 10–14 days. Colonies, as defined to consist of ≥50 cells, were then fixed with 10% neutral buffered formalin (Sigma-Aldrich, St. Louis, MO, USA) for 30 min, stained with crystal violet (0.1% *w*/*v*, Sigma) for 1 h on an orbital shaker, and counted using ImageJ v1.53 (National Institutes of Health, NIH). The relative number of colonies per experimental condition was averaged and normalized to the negative control. Figures represent data obtained from three independent experiments performed in triplicate.

### 2.9. Cell Migration and Invasion Assay

Cell migration and invasion assays were performed in a 24-well plate using polycarbonate inserts with 8μm pores (EMD Millipore, Burlington, MA, USA) coated with (invasion assay) or without (migration assay) Corning Matrigel matrix (growth factor reduced). MDA-MB-231, T47D, and MCF-7 cells were transfected with Y2, Y3, and NTC for 24 h. Transfected cells were harvested via enzyme-free trypsin (Tryp-LE, Thermo Fisher) and re-seeded into the upper chamber of the insert containing 200 µL of serum-free DMEM media at 5 × 10^4^ cells/well (MDA-MB-231 and T47D) or 1 × 10^5^ cells/well (MCF-7). 750 µL of media containing 10% FBS was placed into the lower chamber to induce chemotaxis. After incubation for 24 h, cells migrating through the membrane were fixed in 10% formalin, stained with 0.1% crystal violet, and counted under a microscope. Five visual fields from each insert were randomly selected for cell counting, averaged and presented as mean cells per field. Mean cell counts were normalized to wells containing breast cancer cells transfected with the NTC and presented as percent of control. Data were obtained from two independent experiments performed in triplicate. T47D cells were not assayed for invasion, as they did not readily invade through the Matrigel-coated inserts under our experimental conditions.

### 2.10. RNA Sequencing and Data Processing

Total RNA was extracted from transiently transfected MDA-MB-231 cells using miRNeasy Mini Kit (Qiagen) according to the manufacturer’s protocol. RNA quality was checked by running an aliquot on an Agilent Bioanalyzer (Santa Clara, CA, USA) to confirm integrity, and a Qubit RNA fluorometry assay (Thermo Fisher) was used to measure concentration. Only RNA samples with an RNA Integrity Number (RIN) greater than 8 were used for library preparation. mRNA enrichment and cDNA library preparation utilizing the stranded mRNA (polyA-selected) sample prep kit was performed. RNA-seq was performed at paired-end 150 base pairs (bp) on the Illumina NovaSeq 6000 (San Diego, CA, USA). A minimum of 30M reads were obtained for each sample.

The quality control of RNA-seq raw data was analyzed by FastQC software (https://www.bioinformatics.babraham.ac.uk/projects/fastqc, accessed on 20 February 2020. Babraham Institute, Cambridge, UK). The STAR two-pass method was used for raw data alignment to the human reference genome (hg38). Gene expression levels were determined from aligned BAM files using featureCounts [13]. GENCODE v30 (the National Human Genome Research Institute, Bethesda, MA, USA) was used for coding gene and noncoding RNA annotation in the human genome. DESeq2 [14] was used to quantify the differential expression genes (DEGs) between NTC and *YBEY* k.d. samples at 24 h and 48 h timepoints. DEGs were identified with the false discovery rate (FDR) <0.05 and fold change (FC) >2. DEGs were used for gene ontology (GO) and Kyoto Encyclopedia of Genes and Genomes (KEGG) database pathway analysis by using WebGestalt [15]. GO was classified as cellular component (CC), biological process (BP), and molecular function (MF). Benjamini-Hochberg (BH) adjustment was used to identify enrichment GO categories and KEGG pathway, and only the terms with FDR < 0.05 were considered significantly enriched. MetaCore 5.0 software (Clarivate Analytics, Philadelphia, PA, USA) was also used to identify and validate significantly enriched gene sets and pathways associated with *YBEY* k.d.

### 2.11. Statistical Analysis

UALCAN (http://ualcan.path.uab.edu/, accessed on 9 March 2020) is a publicly available online portal to perform in-depth analyses of The Cancer Genome Atlas (TCGA) gene expression data. We used univariate UALCAN analysis to compare expression of *YBEY* between breast cancer tumor tissues and adjacent normal tissues, as well as various subgroups, based on breast cancer stages. Expression levels were normalized as transcripts per million reads, and a log-rank *p*-value < 0.05 was considered as statistically significant. We further analyzed survival data available through KM Plotter (https://kmplot.com/analysis/, accessed on 11 March 2020), a database which includes publicly available data from TCGA, the Gene Expression Omnibus (GEO), and the European Genome-phenome Archive (EGA) to identify any associations between *YBEY* expression and breast cancer survival by estrogen receptor status.

All data from in vitro experiments were expressed as mean ± SD. A two-tailed paired t-test was used to determine significant difference between two groups. Comparisons between more than two groups were analyzed by one-way ANOVA, followed by Dunnett’s correction for multiple comparisons. Statistics were performed by GraphPad Prism 8.0 (GraphPad Software, San Diego, CA, USA), and *p*-values < 0.05 were considered statistically significant.

## 3. Results

**Figure 1:**
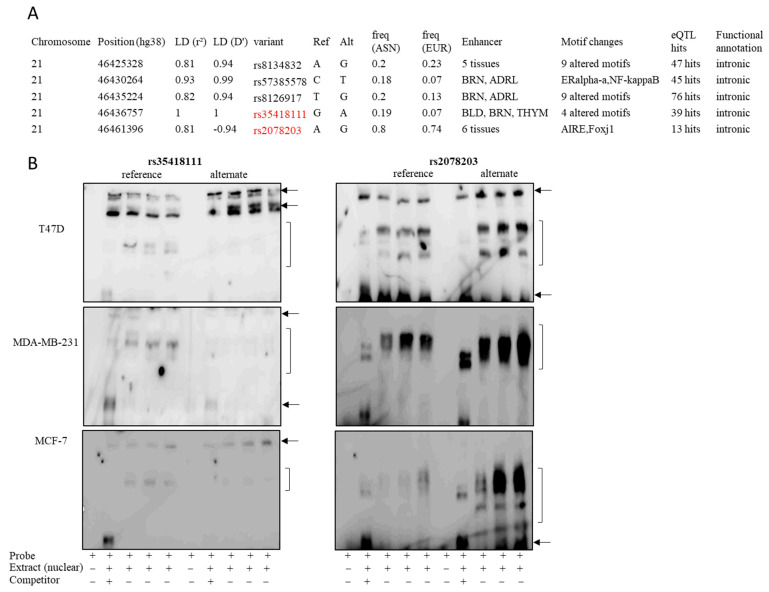
SNPs in high LD of the index SNP (rs35418111) in Asians (rs8134832, rs57385578, rs8126917, and rs2078203) and Europeans (rs57385578) were prioritized using HaploReg v4.1 annotation (**A**) and assessed for differential binding of nuclear proteins by EMSA (**B**). Two SNPs (rs35418111 and rs2078203), indicated in red, were observed to have differences in band intensities between the reference allele sequence and the alternate allele. Differential patterning and band intensities are indicated by parentheses; non-specific binding is indicated by arrows. LD = linear discrimination, ASN= Asian, EUR = European, BLD = blood, BRN = brain, ADRL = adrenal gland, THYM = thymus. Breast cancer cells were incubated with biotin-labeled probes corresponding to the reference allele (lanes 1–5) or the alternate allele (lanes 6–10) in the absence or presence of competitors. Lanes 1 and 6, no nuclear protein extract; lanes 2 and 7, competitor in 200-fold molar excess; lanes 3 and 8 (5 mmol/L MgCl_2_), lanes 4 and 9 (2.5 mmol/L MgCl_2_), and lanes 5 and 10 (1.25 mmol/L MgCl_2_), no competitor.

We first aimed to identify potential causal functional variants for index SNP rs35418111 identified in our previous GWAS. eQTL analysis, using data from GTEx and annotation from HaploReg v4.1 identified *YBEY* as a target gene for rs35418111. SNP rs35418111 and four additional SNPs in high LD with rs35418111 were prioritized based on allele frequency in Asians, motif changes, chromatin alterations, and histone modifications (Figure 1A). Thus, a total of five SNPs, including rs8134832, rs57385578, rs8126917, rs2078203, and rs35418111, were prioritized and assessed by EMSA for changes in DNA-protein interactions based on these criteria. No notable changes in DNA-protein binding were observed for SNPs rs8134832, rs57385578, and rs8126917. We did observe a significant increase in nuclear protein binding for rs35418111 when comparing the reference sequence to the breast cancer risk allele. Conversely, for rs2078203, we observed a significant decrease in protein binding by the reference sequence compared with the alternative sequence (Figure 1B). Differential patterning and band intensities have been indicated; non-specific binding is indicated by black arrows (Figure 1B). Full gel images for rs35418111 and rs2078203 have been provided in Figure S1. eQTL analysis, using data from GTEx, indicated that rs2078203 was associated with *YBEY* expression in breast tissues.

**Figure 2:**
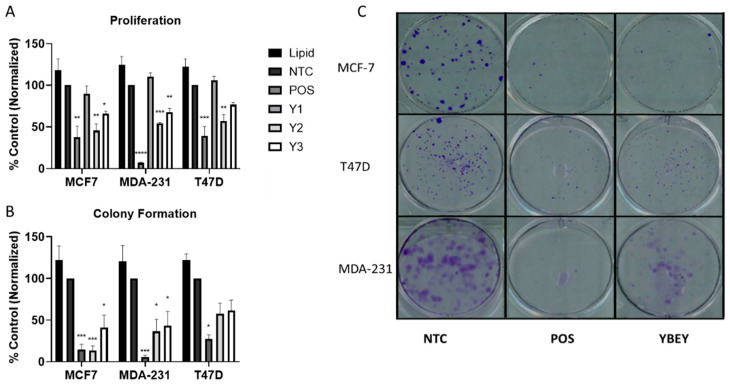
Proliferation and colony formation were assessed following siRNA k.d. of YBEY in breast cancer cells. Both proliferation (**A**) and colony formation (**B**) were reduced following YBEY k.d., when compared to the negative control (NTC). Images for colony formation have been provided for MCF-7 (top), T47D (middle), and MDA-MB-231 (bottom) cell lines (**C**). Statistical significance is denoted by the number of asterisks; * = *p* < 0.05, ** = *p* < 0.01, *** = *p* < 0.001 and **** = *p* < 0.0001).

In vitro functional assay results showing the impact of *YBEY* k.d. on cell proliferation and colony formation are displayed in Figure 2. Following k.d. with three siRNAs targeting *YBEY* mRNA, siRNAs Y2 and Y3 significantly reduced cell proliferation by 54.3% and 33.9%, respectively, in MCF-7 cells (Figure 2A). *YBEY* k.d. resulted in 43.1% and 23.3% decreased proliferation for Y2 and Y3, respectively, in T47D cells. MDA-MB-231 cell proliferation was reduced by 45.6% and 32.2% for Y2 and Y3, respectively. Proliferation was not significantly different from the negative control following treatment with the Y1 siRNA in any cell line. We chose to proceed testing colony forming efficiency with siRNAs Y2 and Y3, as these resulted in a significant decrease in *YBEY* gene expression (Figure S2), as well as significant reductions in proliferation. Y2 and Y3 reduced colony formation in MCF-7 cells by 86.4% and 58.8%, respectively (Figure 2B). In T47D cells, Y2 and Y3 reduced colony forming efficiency by 30.0% and 43.3%, respectively. In MDA-MB-231 cells, colony formation was reduced by 69.6% and 68.1% for Y2 and Y3, respectively. Treatment with both siRNAs resulted in significant decreases in colony formation in MCF-7 and MDA-MB-231 cell lines (Figure 2B). While there was an evident reduction in colony formation in T47D cells, this difference was not significantly different from our negative control group. Representative images are presented (Figure 2C) for all cell lines treated with the NTC, positive control (POS), and *YBEY*-targeting siRNA (Y2).

**Figure 3:**
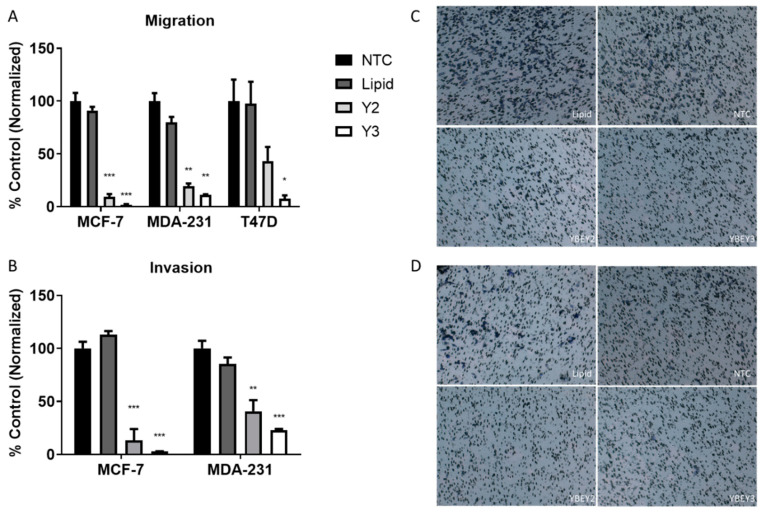
Migration and invasion were evaluated following *YBEY* k.d. in breast cancer cell lines. Migration (**A**) and invasion (**B**) were significantly reduced following *YBEY* k.d., compared to the negative control. Images for migration (**C**) and invasion assays (**D**) were taken of the MDA-MB-231 cell line at 40×. Statistical significance is denoted by the number of asterisks; * = *p* < 0.05, ** = *p* < 0.01, *** = *p* < 0.001 and **** = *p* < 0.0001).

We next performed migration and invasion assays to determine whether *YBEY* k.d. influenced cell mobility and invasion through a basement membrane (Figure 3). Compared with the NTC, Y2 and Y3 reduced migration in MCF-7 cells by 90.4% and 98.2%, respectively. Y2 and Y3 reduced invasion in MCF-7 cells by 86.5% and 97.1%, respectively. In T47D cells, Y2 and Y3 reduced migration by 56.6% and 92.5%, respectively (Figure 3A). Invasion was not measured in T47D cells, as they did not readily invade through the Matrigel coated inserts in our culture conditions. Y2 and Y3 significantly reduced MDA-MB-231 migration by 80.6% and 88.6%, respectively. Y2 and Y3 resulted in a decrease in MDA-MB-231 cell invasion by 59.3% and 77.1%, respectively (Figure 3B). Representative images are provided to illustrate MDA-MB-231 cell migration (Figure 3C) and invasion (Figure 3D), following treatment with the vehicle (lipid), NTC, and siRNAs targeting *YBEY* (Y2 and Y3).

**Figure 4:**
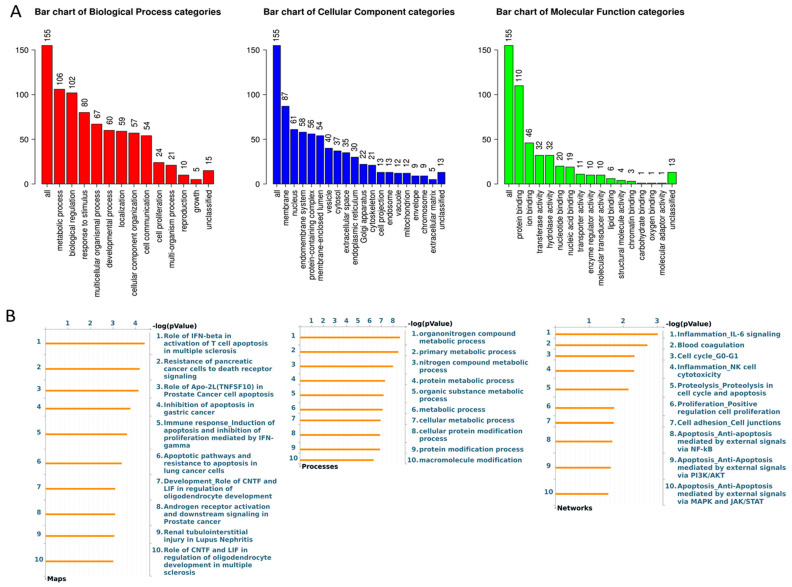
RNA sequencing was performed following siRNA mediated k.d. of *YBEY*. GSEA was performed to demonstrate Biological Processes, Cellular Components, and Molecular Functions of the DEGs (**A**). WebGestalt was used to identify enriched pathways in *YBEY* k.d. samples (**B**). Pathway enrichment was also performed using MetaCore analysis tools to identify gene maps, processes, and networks associated with *YBEY* k.d.

RNA-seq and subsequent pathway analysis were performed to identify genes and gene networks associated with the *YBEY* gene. WebGestalt and Metacore analysis tools were used to determine cellular processes and pathways significantly up- or down-regulated following transient *YBEY* k.d. at 24 and 48 h (Figure 4). At 24 h, we identified 45 DEGs following *YBEY* k.d. At 48 h, we identified 155 DEGs (Table S4). Of note, all genes up- or down-regulated at the 24 h timepoint were also differentially expressed at the 48h timepoint. Due to our small sample size per condition (*n* = 2), we performed qPCR to confirm *YBEY* k.d., as well as to validate that mRNAs identified by RNA-seq could be replicated (Figure S3). Principle component analysis showed consistent clustering of each replicate for our treatment conditions. GSEA analysis showed several DEGs identified in our analysis were involved in metabolic processes and biological regulation, the majority of which are protein binding (Figure 4A). Additionally, we used Metacore pathway analysis to identify the top 10 enriched gene maps, processes, and networks at the 48h timepoint (Figure 4B). Several gene networks were identified using both tools including immune response and the metabolism/modification of proteins and macronutrients; however, many networks did not reach the threshold for significance using the MetaCore 5.0 software.

**Figure 5:**
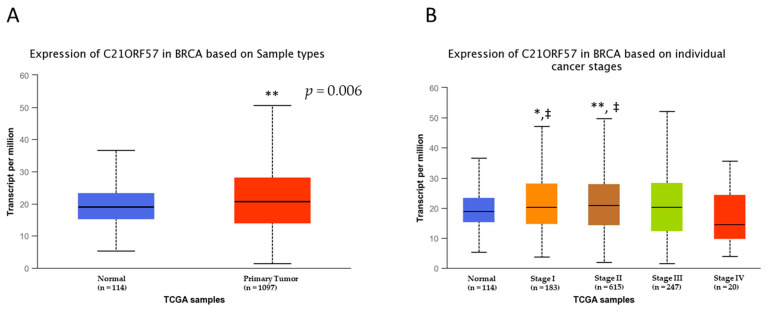
Expression of *YBEY* (*C21ORF57*) in TCGA breast tumor datasets: *YBEY* expression was compared in normal tissues and tumor tissues of breast cancer patients from TCGA (**A**). Expression of *YBEY* was also compared across stages at diagnosis in the TCGA dataset (**B**). An asterisk (* = *p* < 0.05, ** = *p* < 0.01) denotes the level of significance from normal tissues and the double dagger (‡, *p* < 0.05) represents a statistical difference from stage IV disease.

Finally, we found that in the TCGA breast cancer (TCGA-BRCA) dataset, *YBEY* expression was significantly increased in tumors compared with normal tissues (Figure 5A). Interestingly, *YBEY* expression at Stage IV disease was significantly decreased from early-stage disease (i.e., Stage I and Stage II). Univariate analysis revealed that *YBEY* expression was positively associated with overall survival using the same dataset (*p* = 0.035, Figure S4A); however, results derived from KM Plotter showed that this association may be dependent upon estrogen receptor status (Figure S4B,C).

## 4. Discussion

Human *YBEY* is an uncharacterized gene with no known function; however, it is predicted to function similarly to its bacterial homologues [16]. Our study demonstrates that several genetic variants associated with *YBEY* expression may be functional via increasing or decreasing nuclear protein binding. We also show that k.d. of *YBEY* results in significant reductions in breast cancer cell proliferation, colony formation, migration, and invasion, in vitro. Pathway analysis from RNA-seq data shows several pathways, including metabolic and immune processes, were affected by siRNA-mediated k.d. of *YBEY*. Finally, we show that *YBEY* expression is higher in early-stage tumor tissues than adjacent normal tissues in TCGA breast cancer database. However, *YBEY* expression was significantly reduced in late-stage disease compared with early-stage, suggesting a potential molecular phenotype switch in metastatic disease compared with initial stages.

*YBEY* was initially discovered to function as an endoribonuclease in *E. Coli* [4]. Subsequent studies suggest that it is critical for bacterial cell growth due to its significant function in rRNA maturation and ribosome biogenesis [6]. We showed that cellular functions, including proliferation, colony formation, and invasion/migration, were significantly reduced following *YBEY* mRNA k.d. in human breast cancer cell lines. These data suggest that *YBEY* may have a global function in mammalian systems outside of rRNA maturation, as characterized in bacteria.

While *YBEY* k.d. may disrupt proper rRNA maturation and ribosomal biogenesis, migration and invasion of cancer cell lines depend on several factors, including chemokine/cytokine signaling, cytoskeletal rearrangements, and matrix metalloproteases [17,18]. To test whether *YBEY* may regulate genes involved in these processes, we performed RNA-seq following transient *YBEY* k.d. Our pathway analysis provides evidence that *YBEY* may have functions outside of proliferation and ribosomal biogenesis. The top pathways identified in our RNA-seq analysis included metabolic processes and immune responses. Interestingly, ribosomal biogenesis has been reported to play a critical role in immune response through interferon signaling [19] and other signaling pathways [20]. While the power and, thus, interpretation of our RNA-seq analysis is limited due to the small sample size, this is the first study to indicate a potential function of human *YBEY*. Transient k.d. using siRNAs greater than 23 bp in length has been reported to induce interferon signaling [21]; however, the siRNAs used in this study were less than 20 bp, which should minimize any innate immune signaling in our breast cancer cell lines. Further, we observed a global decrease in genes associated with immune response. Further mechanistic studies utilizing stable transfections (i.e., CRISPR) would provide more robust evidence for this association. Additionally, it was recently discovered that *YBEY* expression may be significantly altered by DNA methylation [22]. However, we did not consider the impact of epigenetics when assessing the associations between SNPs and *YBEY* expression. Further studies elucidating the mechanisms by which *YBEY* regulates the cellular functions observed in our in vitro screens are warranted.

Our previous GWAS and subsequent eQTL analysis suggest that *YBEY* expression may be inversely associated with breast cancer risk [3]. Data from TCGA showed that, compared to normal adjacent tissue, breast cancers had a significantly increased expression of *YBEY*. However, metastatic disease (i.e., Stage IV) had significantly decreased *YBEY* expression compared with Stage I and II breast cancers. This trend may underpin the disparities seen in survival analysis derived from TCGA when grouped by median *YBEY* expression. While higher *YBEY* expression appears to be beneficial in overall survival analyses, these data may be skewed due to the higher number of earlier stage breast cancers in TCGA or potentially confounded by estrogen receptor status, as observed in our survival analysis. Additionally, our study utilized breast cancer cell lines that had already undergone oncogenic transformation; consequently, using in vitro functional assays to test cancer cell lines may not completely recapitulate breast cancer risk, but may illustrate that *YBEY* is involved in disease progression, as suggested by the trend in *YBEY* expression by cancer stage.

## 5. Conclusions

Overall, our study has provided preliminary evidence that the human *YBEY* gene, and SNPs associated with its expression, may have a potential causal function in breast cancer risk. In vitro functional assays showed a significant reduction in several cellular processes associated with cancer progression including proliferation, clonal capacity, and migration/invasion. RNA-seq analysis provided additional clues to further roles of *YBEY* in immunological or metabolic processes as well. Data derived from TCGA provided evidence of differential *YBEY* expression depending on cancer stage, with metastatic disease having significantly lower expression compared with earlier stages. These studies provide the first evidence that *YBEY* may contribute to cancer risk and/or cancer progression. Further studies are necessary to shed light on this uncharacterized gene and its involvement in carcinogenesis.

## Data Availability

The datasets generated during and/or analyzed during the current study are available from the corresponding author on reasonable request.

## Supplementary Materials

The following are available online at https://www.mdpi.com/article/10.3390/cancers13092037/s1, Figure S1. EMSA for reference and risk alleles, Figure S2. qPCR analysis of *YBEY* expression following knockdown of *YBEY* mRNA in breast cancer cell lines, Figure S3. qPCR validation of *YBEY* k.d. and RNA sequencing, Figure S4. Unadjusted overall survival (OS) curves for *YBEY*-high and *YBEY*-low breast cancers, Table S1. Oligo sequences used for gel shift assays, Table S2. Sequences for siRNAs targeting *YBEY* mRNA, Table S3. Primer sequences used for qPCR validation of genes identified in RNA-seq analysis, Table S4. Full list of DEG (si*YBEY* vs. NT) from RNA-seq of MDA-MB-231 cells at the 48h time point.
