# Supplementary material for: Functional Genomic Analyses of the 21q22.3 Locus Identifying Functional Variants and Candidate Gene YBEY for Breast Cancer Risk
Source: Cancers (Basel). 2021 Apr 23;13(9):2037. doi: 10.3390/cancers13092037 (PMC8122893; doi:10.3390/cancers13092037)
Supplement: Supplementary file 1 [file cancers-13-02037-s001.zip › cancers-1133081-supplementary.pdf]

cancers

MDPI

Supplementary Materials: Functional Genomic Analyses of the 21q22.3 Locus Identifying Functional Variants and Candidate Gene *YBEY* for Breast Cancer Risk

Chris Shidal, Xiang Shu[1], Jie Wu, Jifeng Wang, Shuya Huang, Jirong Long, Joshua A. Bauer, Jie Ping, Xingyi Guo, Wei Zheng, Xiao-Ou Shu, and Qiuyin Cai

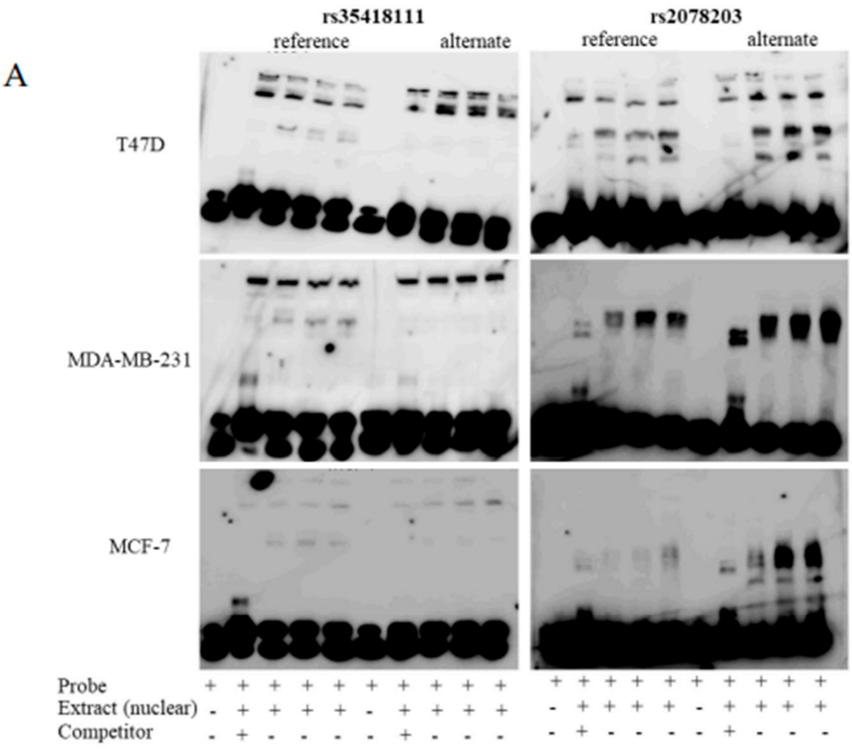

B

	rs35418111				rs2078203			
[C] $MgCl_2$ (mmol)	5	2.5	1.25	Avg	5	2.5	1.25	Avg
T47D	10.78	2.02	3.05	5.28	0.61	0.48	0.69	0.60
MDA-231	5.96	3.00	5.60	4.85	0.45	0.64	0.38	0.49
MCF-7	4.25	2.51	2.34	3.04	0.22	0.07	0.15	0.14

Figure S1. EMSA for reference and risk alleles. Breast cancer cell line lysates were incubated with biotin-labeled probes corresponding to the reference allele (lanes 1–5) or the risk allele (lanes 6–10) in the absence or presence of competitors (Supplemental Figure 1A). These images represent unaltered, full gel images for rs35418111 and rs2078203 in three breast cancer cell lines. Differential binding intensities between reference and risk alleles were quantitated for each condition and have been provided in Supplemental Figure 1B. Densitometric analyses of specific bands were performed and values represent normalized ratios of reference to risk allele band intensities.

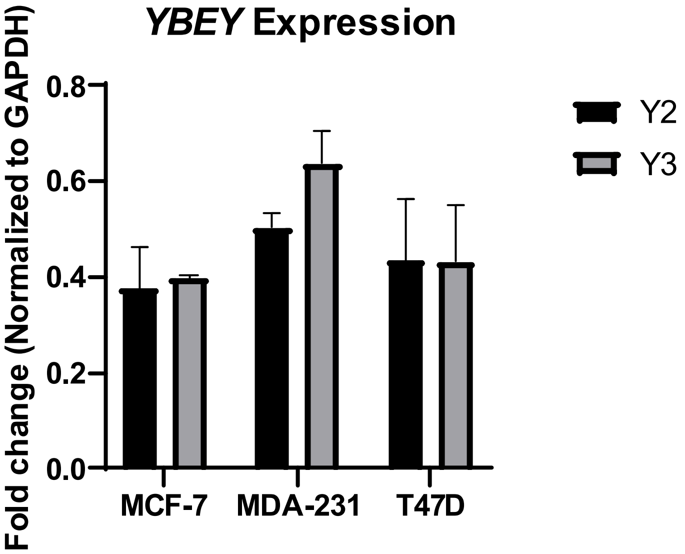

Figure S2. qPCR analysis of *YBEY* expression following knockdown of *YBEY* mRNA in breast cancer cell lines. Fold change was calculated by normalizing siRNAs Y2 and Y3 targeting *YBEY* mRNA to the non-targeting control (NTC) using GAPDH as a housekeeping gene.

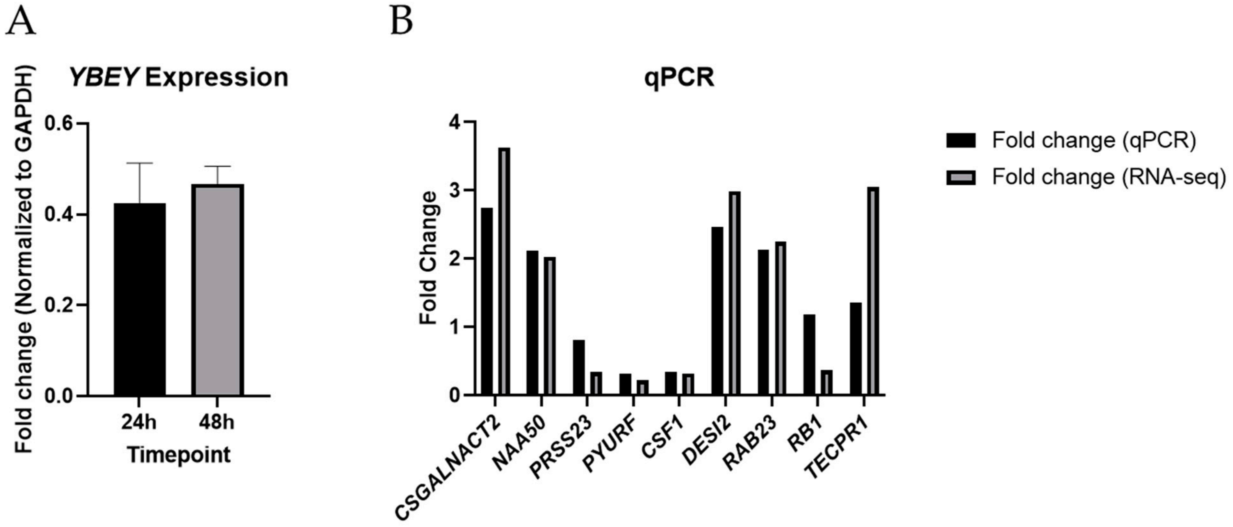

Figure S3. qPCR validation of *YBEY* knockdown (k.d.) and RNA sequencing. qPCR was performed to confirm adequate *YBEY* mRNA k.d., prior to RNA sequencing. qPCR was also performed post-sequencing to validate several of the DEGs identified in RNA-seq analysis following *YBEY* k.d. in the MDA-MB-231 cell line. Fold change was calculated using the ΔΔCt method and has been normalized to the NTC using GAPDH as a reference gene.

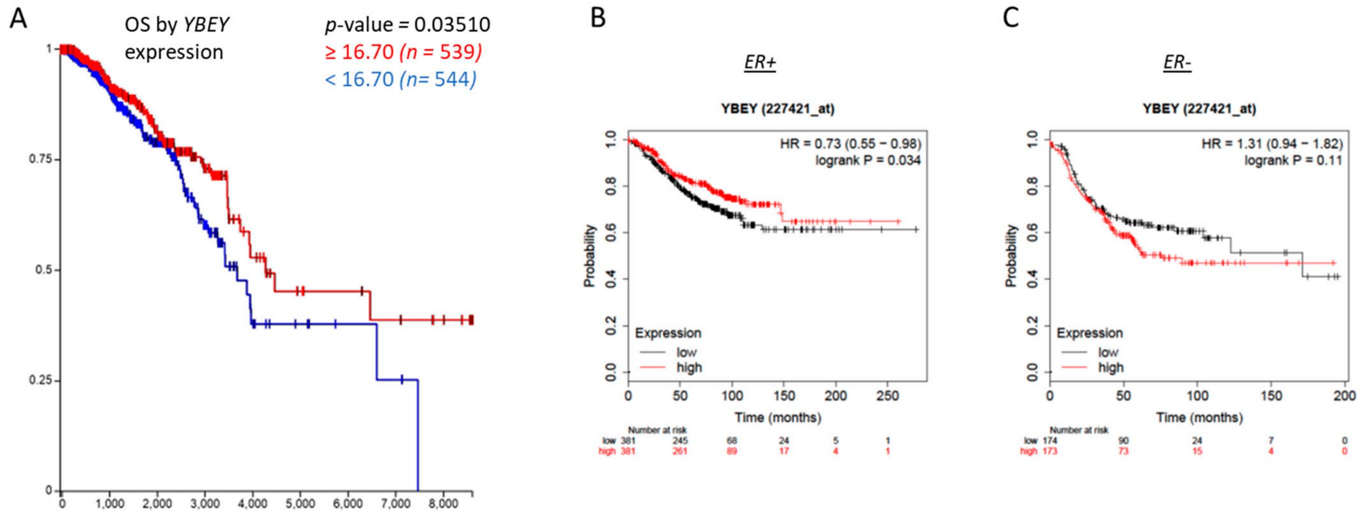

Figure S4. Unadjusted overall survival (OS) curves for *YBEY*-high (red) and *YBEY*-low (blue) breast cancers in the TCGA database using UCSC Xena online software (https://xena.ucsc.edu/. Accessed on 11 March 2020) (**A**). Survival curves using KM Plotter show disparities in survival depending on ER status in breast cancer patients (**B,C**).

Table S1. Oligo sequences used for gel shift assays which correspond to reference and risk alleles (mutation sites are highlighted in red).

**rs35418111**	
Forward ref : CCGTGCTCGGTGGCGCGCCTCCCGGCTGG	Biotin: 5Biosg/ CCGTGCTCGGTGGCGCGCCTCCCGGCTGG
Reverse ref: CCAGCCGGGAGGCGCGCCACCGAGCACGG	
Forward alt: CCGTGCTCGGTGGCACGCCTCCCGGCTGG	Biotin: 5Biosg/ CCGTGCTCGGTGGCACGCCTCCCGGCTGG
Reverse alt: CCAGCCGGGAGGCGTGCCACCGAGCACGG	
**rs57385578**	
Forward ref : TCCTGGGACACTGGCGGGAGTCCCCCCGT	Biotin: 5Biosg/ TCCTGGGACACTGGCGGGAGTCCCCCCGT
Reverse ref: ACGGGGGGACTCCCGCCAGTGTCCCAGGA	
Forward alt: TCCTGGGACACTGGTGGGAGTCCCCCCGT	Biotin: 5Biosg/ TCCTGGGACACTGGTGGGAGTCCCCCCGT
Reverse alt: ACGGGGGGACTCCCACCAGTGTCCCAGGA	
**rs8130538**	
Forward ref : AAAATGTATCCAGGTGTGGTGACACATAC	Biotin: 5Biosg/ AAAATGTATCCAGGTGTGGTGACACATAC
Reverse ref: GTATGTGTCACCACACCTGGATACATTTT	
Forward alt: AAAATGTATCCAGGCGTGGTGACACATAC	Biotin: 5Biosg/ AAAATGTATCCAGGCGTGGTGACACATAC
Reverse alt: GTATGTGTCACCACGCCTGGATACATTTT	
**rs8126917**	
Forward ref: TGTTGTTTTTTTTGTTTTGGTTTGGTTTT	Biotin: 5Biosg/ TGTTGTTTTTTTTGTTTTGGTTTGGTTTT
Reverse ref: AAAACCAAACCAAAACAAAAAAAACAACA	
Forward alt: TGTTGTTTTTTTTGGTTTGGTTTGGTTTT	Biotin: 5Biosg/ TGTTGTTTTTTTTGGTTTGGTTTGGTTTT
Reverse alt: AAAACCAAACCAAACCAAAAAAAACAACA	
**rs2078203**	
Forward ref: ATCTACTGTACTGCATTTGTTAAGGCTCT	Biotin: 5Biosg/ ATCTACTGTACTGCATTTGTTAAGGCTCT
Reverse ref: AGAGCCTTAACAAATGCAGTACAGTAGAT	
Forward alt: ATCTACTGTACTGCGTTTGTTAAGGCTCT	Biotin: 5Biosg/ ATCTACTGTACTGCGTTTGTTAAGGCTCT

Reverse alt: AGAGCCTTAACAAACGCAGTACAGTAGAT

Table S2. Sequences for siRNAs targeting *YBEY* mRNA.

siRNA	siRNA Sequence	Catalogue no.
si*YBEY*1	CCAGAUGACUACAAUUUGG	D-031405-08-0002
si*YBEY*2	ACAAUGACGUCCUGACUGU	D-031405-09-0002
si*YBEY*3	GAAAUGUCCCAACCGAUGU	D-031405-10-0002

Table S3. Primer sequences used for qPCR validation of genes (in italics) identified in RNA-seq analysis as well as amplicon lengths.

Sequence (5′- 3′)	Template Strand
Forward primer	GCCCGGGGAAAGTGAAAGTT
Reverse primer	CACCGCTGTCCTGGGATG
Product length	73
DESI2	-
Forward primer	CCGGGCTGTACGCTTAGTG
Reverse primer	AACTGGTTAGCCCCCATCCT
Product length	137
MAPK6	-
Forward primer	ATGTGGCATCGCAGTCTCTT
Reverse primer	ACCCAAGAGTGAAACAGGAGC
Product length	140
RB1	-
Forward primer	TTGTAACGGGAGTCGGGAGA
Reverse primer	CAGCGAGCTGTGGAGGAG
Product length	83
TECPR1	-
Forward primer	AAATCAGCACATCTGGGCCT
Reverse primer	TTCCACCAGCTAACTTCTGCT
Product length	142
RAB23	-
Forward primer	GGTCCACAGATGAGGGGAGT
Reverse primer	CCAGGGCTCAGATTGAGTGG
Product length	84
PYURF	-
Forward primer	TCTCCAAGAAGCCGCTCAGA
Reverse primer	ATCCTAGCTGCCTGTGGTATC
Product length	118
PRSS23	-
Forward primer	ATGGGCCAATGGGGATGTTG
Reverse primer	AGCTTTGTGGGAGCATTTGG
Product length	139
NAA50	-
Forward primer	AAGAGGATAGAGCCCGCAGA
Reverse primer	GCGACAAGCAAGTGCAAGAA
Product length	131
CSGALNACT2	-
Forward primer	ACCCTGTGGTGTTCAGTCTT
Reverse primer	CCAAAGCCAAAATCTCGCCA
Product length	124
–	(1)

Table S4. Full list of DEG (si*YBEY* vs. NT) from RNA-seq of MDA-MB-231 cells at the 48h time point. Genes names, transcript counts per replicate sample, adjusted *p*-values, and fold change have been provided in each column. Red text indicates down-regulated genes and green text indicates up-regulated genes in samples treated with *YBEY*-targeting siRNA compared to the NT control.

Ensemble ID	Chromosome	Gene Name	*YBEY1*	*YBEY2*	NT1	NT2	log2 Fold Change	Adjusted *p*-value	Fold Change
ENSG00000168092.14	chr11	*PAFAH1B2*	5066	3279	874	821	-2.248322426	1.26×10^{-20}	0.210468696
ENSG00000107798.18	chr10	*LIPA*	1517	1477	325	311	−2.209202737	2.93×10^{-19}	0.216253781
ENSG00000084090.13	chr2	*STARD7*	4808	3333	1020	908	−2.031968299	3.81×10^{-18}	0.244521241
ENSG00000145337.5	chr4	*PYURF*	2645	1624	438	461	−2.191319504	3.77×10^{-17}	0.218951084
ENSG00000111652.10	chr12	*COPS7A*	1620	1460	384	393	−1.955293821	6.06×10^{-16}	0.257868272
ENSG00000169826.8	chr10	*CSGALNACT2*	525	492	1954	1675	1.862053042	1.79×10^{-14}	3.635246116
ENSG00000136159.4	chr13	*NUDT15*	921	686	175	203	−2.042763094	1.41×10^{-13}	0.242698468
ENSG00000067955.15	chr16	*CBFB*	1878	1302	463	364	−1.898939559	3.01×10^{-13}	0.268140388
ENSG00000084070.12	chr1	*SMAP2*	589	652	134	137	−2.176129599	3.16×10^{-13}	0.221268563
ENSG00000035687.10	chr1	*ADSS2*	2425	1700	584	599	−1.75440303	9.87×10^{-13}	0.296395812
ENSG00000150687.12	chr11	*PRSS23*	18334	14728	5543	5407	−1.555973925	1.08×10^{-12}	0.34009886
ENSG00000164466.13	chr5	*SFXN1*	3262	2752	1032	916	−1.592731109	5.62×10^{-12}	0.331543228
ENSG00000069956.12	chr15	*MAPK6*	2013	2026	655	499	−1.787323964	5.69×10^{-12}	0.289708925
ENSG00000171867.17	chr20	*PRNP*	12435	9485	3821	3623	−1.516976392	1.11×10^{-11}	0.349417461
ENSG00000177733.7	chr5	*HNRNPA0*	4893	3498	1438	1330	−1.555206992	4.26×10^{-11}	0.340279704
ENSG00000121644.19	chr1	*DESI2*	685	621	1979	1840	1.578064617	4.27×10^{-11}	2.98569049
ENSG00000135535.17	chr6	*CD164*	3705	2922	1197	1164	−1.44961077	5.11×10^{-10}	0.366120188
ENSG00000012660.14	chr6	*ELOVL5*	6328	5070	2154	2015	−1.41327441	5.52×10^{-10}	0.37545856
ENSG00000147601.14	chr8	*TERF1*	692	422	138	143	−1.931642895	7.43×10^{-10}	0.262130495
ENSG00000196743.9	chr5	*GM2A*	1159	1106	390	347	−1.593751892	8.87×10^{-10}	0.331308726
ENSG00000168297.16	chr3	*PXK*	2281	2174	833	690	−1.52374067	9.49×10^{-10}	0.347783003
ENSG00000184371.14	chr1	*CSF1*	7218	9345	2820	2384	−1.664995288	4.52×10^{-09}	0.315345382
ENSG00000116473.14	chr1	*RAP1A*	2383	1582	695	665	−1.493869233	8.31×10^{-09}	0.355059021
ENSG00000178691.11	chr17	*SUZ12*	2753	2167	1021	891	−1.325847363	7.61×10^{-08}	0.398914823
ENSG00000166803.13	chr15	*PCLAF*	1090	524	204	220	−1.857941121	7.66×10^{-08}	0.275869694
ENSG00000188725.8	chr5	*SMIM15*	716	436	1571	1514	1.475507199	1.27×10^{-07}	2.780813901
ENSG00000139687.15	chr13	*RB1*	1752	1756	728	567	−1.417042975	2.03×10^{-07}	0.374479078
ENSG00000085998.14	chr1	*POMGNT1*	1924	1842	773	703	−1.325345614	2.12×10^{-07}	0.399053584
ENSG00000196663.16	chr14	*TECPR2*	333	421	1277	1011	1.609185497	2.12×10^{-07}	3.050795543
ENSG00000126970.16	chrX	*ZC4H2*	348	328	88	106	−1.769803893	2.89×10^{-07}	0.293248596
ENSG00000108960.9	chr17	*MMD*	640	419	160	172	−1.621743939	2.89×10^{-07}	0.324942434
ENSG00000100522.10	chr14	*GNPNAT1*	503	494	1313	1205	1.36173113	2.89×10^{-07}	2.569933681
ENSG00000165244.7	chr9	*ZNF367*	816	638	1843	1621	1.290473837	3.66×10^{-07}	2.446083812
ENSG00000048140.18	chr5	*TSPAN17*	2571	1906	883	906	−1.279304003	4.18×10^{-07}	0.411994219
ENSG00000128524.5	chr7	*ATP6V1F*	2430	1454	698	690	−1.427757261	4.34×10^{-07}	0.371708282
ENSG00000162654.9	chr1	*GBP4*	260	354	86	68	−1.991607787	4.69×10^{-07}	0.251458498
ENSG00000023445.15	chr11	*BIRC3*	2949	2377	1181	997	−1.254356801	4.69×10^{-07}	0.41918041
ENSG00000156136.10	chr4	*DCK*	1239	829	411	339	−1.416379442	7.43×10^{-07}	0.374651351
ENSG00000148110.16	chr9	*MFSD14B*	3005	2593	1245	1116	−1.21320074	7.44×10^{-07}	0.431310655
ENSG00000163235.16	chr2	*TGFA*	2305	2154	990	824	−1.271463307	7.51×10^{-07}	0.414239402
ENSG00000101856.10	chrX	*PGRMC1*	1803	1288	567	627	−1.324790932	7.85×10^{-07}	0.39920704
ENSG00000118242.16	chr2	*MREG*	328	239	77	86	−1.75314981	1.21×10^{-06}	0.296653393
ENSG00000107959.16	chr10	*PITRM1*	2942	3052	1320	1142	−1.263731876	1.54×10^{-06}	0.416465278
ENSG00000156802.13	chr8	*ATAD2*	4141	4228	1822	1704	−1.224843966	1.54×10^{-06}	0.427843784
ENSG00000198648.11	chr2	*STK39*	994	932	401	359	−1.314279606	1.61×10^{-06}	0.402126243
ENSG00000023909.10	chr1	*GCLM*	3849	2664	1365	1331	−1.224847535	1.63×10^{-06}	0.427842725
ENSG00000138600.10	chr15	*SPPL2A*	1377	1058	489	506	−1.249323498	2.90×10^{-06}	0.420645409
ENSG00000162434.13	chr1	*JAK1*	2322	2885	1078	896	−1.391456371	2.96×10^{-06}	0.381179815
ENSG00000186106.11	chr8	*ANKRD46*	547	396	171	162	−1.457892319	3.22×10^{-06}	0.364024557
ENSG00000122705.17	chr9	*CLTA*	9121	5807	2718	3230	−1.271251286	5.05×10^{-06}	0.414300284

ENSG00000186480.13	chr7	*INSIG1*	2312	1891	941	911	−1.14539033	5.63 × 10−06	0.452067363
ENSG00000108953.17	chr17	*YWHAE*	22515	16007	8122	8762	−1.142238149	5.90 × 10−06	0.453056176
ENSG00000089902.10	chr14	*RCOR1*	1644	1687	750	642	−1.238132069	6.37 × 10−06	0.423921173
ENSG00000069869.16	chr15	*NEDD4*	540	593	1602	1209	1.325871052	8.88 × 10−06	2.506841969
ENSG00000164983.7	chr8	*TMEM65*	952	699	359	306	−1.270291592	1.08 × 10−05	0.414575972
ENSG00000120889.13	chr8	*TNFRSF10B*	3113	3775	1506	1300	−1.28564219	1.10 × 10−05	0.410188176
ENSG00000120805.14	chr12	*ARL1*	2981	2223	1112	1180	−1.138734103	1.10 × 10−05	0.454157905
ENSG00000068383.19	chr10	*INPP5A*	461	407	1009	942	1.200236932	1.10 × 10−05	2.297774039
ENSG00000084764.12	chr2	*MAPRE3*	272	193	71	71	−1.665966241	1.92 × 10−05	0.315133222
ENSG00000170348.9	chr14	*TMED10*	6065	4561	2364	2517	−1.078447561	2.35 × 10−05	0.473538109
ENSG00000117862.13	chr1	*TXNDC12*	189	169	59	27	−2.034497894	2.58 × 10−05	0.244092878
ENSG00000141696.13	chr17	*P3H4*	450	328	134	148	−1.418620751	2.67 × 10−05	0.37406976
ENSG00000182362.14	chr21	*YBEY*	344	203	92	76	−1.649335716	3.09 × 10−05	0.318786907
ENSG00000166471.11	chr11	*TMEM41B*	1460	858	466	454	−1.275790581	3.68 × 10−05	0.412998778
ENSG00000140511.11	chr15	*HAPLN3*	205	189	65	55	−1.686882993	4.33 × 10−05	0.31059726
ENSG00000081923.14	chr18	*ATP8B1*	293	329	861	669	1.313401304	4.33 × 10−05	2.485267776
ENSG00000165169.11	chrX	*DYNLT3*	1413	919	507	478	−1.192690005	4.69 × 10−05	0.437486376
ENSG00000119231.11	chr3	*SENP5*	1449	1266	661	591	−1.085159455	6.51 × 10−05	0.47134017
ENSG00000173852.14	chr7	*DPY19L1*	2037	1826	974	825	−1.073501891	6.51 × 10−05	0.47516422
ENSG00000058272.19	chr12	*PPP1R12A*	3111	1930	1117	1091	−1.136516703	8.45 × 10−05	0.454856475
ENSG00000121579.13	chr3	*NAA50*	2305	1715	3929	3985	1.02088779	8.62 × 10−05	2.029167262
ENSG00000072415.9	chr14	*MPP5*	2298	2166	1148	949	−1.064599783	0.000103573	0.478105271
ENSG00000182253.15	chr15	*SYNM*	1059	873	454	431	−1.090223354	0.000116473	0.469688653
ENSG00000136045.12	chr12	*PWP1*	2728	1911	1048	1100	−1.062801101	0.000119277	0.478701722
ENSG00000119714.11	chr14	*GPR68*	1977	1618	855	863	−1.027700146	0.000120382	0.490491436
ENSG00000128923.11	chr15	*MINDY2*	755	556	300	263	−1.177664081	0.000122938	0.442066685
ENSG00000078124.12	chr11	*ACER3*	1225	845	463	455	−1.125413053	0.000122938	0.458370768
ENSG00000170961.7	chr8	*HAS2*	262	225	579	506	1.188173705	0.000137285	2.278641094
ENSG00000025039.15	chr6	*RRAGD*	1211	753	391	433	−1.197237528	0.000156258	0.436109545
ENSG00000178105.11	chr11	*DDX10*	2307	1504	824	887	−1.102480161	0.000157756	0.465715188
ENSG00000189159.16	chr17	*JPT1*	8797	5015	2774	3143	−1.160638543	0.000158621	0.447314508
ENSG00000118515.11	chr6	*SGK1*	455	308	859	771	1.141708006	0.000169417	2.206420866
ENSG00000119541.10	chr18	*VPS4B*	562	525	1196	1044	1.070400523	0.000175122	2.100016296
ENSG00000253276.3	chr7	*CCDC71L*	627	462	1077	1155	1.080074182	0.000177796	2.114144786
ENSG00000137801.11	chr15	*THBS1*	21795	22483	####	9188	−1.052836554	0.000179586	0.48201951
ENSG00000117222.14	chr1	*RBBP5*	807	796	377	348	−1.120089239	0.000197658	0.460065367
ENSG00000138182.14	chr10	*KIF20B*	4357	2879	1679	1736	−1.03176116	0.000249716	0.489112703
ENSG00000101363.12	chr20	*MANBAL*	2198	1481	820	883	−1.0604447	0.000257296	0.479484239
ENSG00000140612.14	chr15	*SEC11A*	3069	1643	906	1039	−1.210521465	0.00026003	0.4321124
ENSG00000169976.7	chr6	*SF3B5*	2536	1442	723	920	−1.211704096	0.000281065	0.431758326
ENSG00000132964.12	chr13	*CDK8*	642	477	253	237	−1.14918183	0.000284504	0.450880859
ENSG00000168386.18	chr3	*FILIP1L*	119	98	268	270	1.346830235	0.000289929	2.543526695
ENSG00000103196.12	chr16	*CRISPLD2*	424	549	197	186	−1.336550145	0.000291061	0.395966382
ENSG00000064393.16	chr7	*HIPK2*	3855	4134	2154	1540	−1.098190094	0.000349489	0.467102123
ENSG00000174010.9	chrX	*KLHL15*	699	634	330	277	−1.106447129	0.000367366	0.464436374
ENSG00000101935.10	chrX	*AMMECR1*	426	398	950	766	1.084434341	0.000371893	2.120543887
ENSG00000131015.5	chr6	*ULBP2*	427	435	180	185	−1.215284954	0.000403739	0.430688004
ENSG00000164073.10	chr4	*MFSD8*	801	658	332	346	−1.067904939	0.000413015	0.477011205
ENSG00000173432.12	chr11	*SAA1*	696	340	151	209	−1.452021399	0.000421945	0.365508941
ENSG00000145623.13	chr5	*OSMR*	1879	2307	1059	836	−1.135507523	0.000492413	0.455174764
ENSG00000125611.15	chr2	*CHCHD5*	336	213	91	109	−1.4023313	0.000520784	0.378317311
ENSG00000213463.5	chr14	*SYNJ2BP*	698	647	345	264	−1.117364647	0.000520784	0.460935041
ENSG00000108406.10	chr17	*DHX40*	975	864	481	399	−1.033749148	0.000548141	0.488439186
ENSG00000198933.9	chr17	*TBKBP1*	331	325	147	123	−1.256699109	0.000631691	0.418500395
ENSG00000160712.13	chr1	*IL6R*	46	31	127	121	1.731972472	0.000645974	3.321816712
ENSG00000240065.8	chr6	*PSMB9*	1423	794	423	511	−1.183205562	0.000683842	0.440371936
ENSG00000162396.6	chr1	*PARS2*	214	220	90	71	−1.409730277	0.000895417	0.376382048

ENSG00000205133.12	chr8	*TRIQK*	574	460	243	236	−1.072010144	0.001010184	0.475655794
ENSG00000160131.13	chrX	*VMA21*	2135	1124	688	730	−1.13497831	0.001040946	0.455341762
ENSG00000163293.12	chr4	*NIPAL1*	209	216	523	411	1.156404449	0.001044313	2.229012101
ENSG00000182093.16	chr21	*GET1*	414	240	103	140	−1.36649533	0.001100575	0.387832247
ENSG00000113638.14	chr5	*TTC33*	447	379	170	195	−1.141167622	0.001100575	0.453392483
ENSG00000140465.14	chr15	*CYP1A1*	622	634	271	309	−1.088883075	0.001143228	0.470125201
ENSG00000115827.14	chr2	*DCAF17*	516	419	238	184	−1.11363608	0.001231585	0.462127843
ENSG00000067141.17	chr15	*NEO1*	199	247	84	81	−1.422224597	0.001243925	0.373136502
ENSG00000163875.15	chr1	*MEAF6*	1099	708	397	441	−1.054897632	0.001256946	0.481331374
ENSG00000111224.14	chr12	*PARP11*	167	145	59	49	−1.4989636	0.001347725	0.353807467
ENSG00000197147.13	chr1	*LRRC8B*	112	116	37	31	−1.723594879	0.001497777	0.302793286
ENSG00000040199.18	chr16	*PHLPP2*	553	700	289	275	−1.141826525	0.001830747	0.453185458
ENSG00000166831.9	chr15	*RBPMS2*	77	89	20	21	−1.999289439	0.001897284	0.250123161
ENSG00000119285.11	chr1	*HEATR1*	334	557	1313	904	1.303480617	0.002081164	2.468236467
ENSG00000107443.16	chr10	*CCNJ*	542	417	232	224	−1.032028195	0.002346752	0.489022179
ENSG00000164400.6	chr5	*CSF2*	542	366	200	214	−1.083352358	0.002528712	0.471930933
ENSG00000197249.14	chr14	*SERPINA1*	736	614	274	362	−1.045524448	0.002551645	0.484468762
ENSG00000112183.15	chr6	*RBM24*	155	112	294	281	1.149665758	0.002841163	2.218624877
ENSG00000112210.12	chr6	*RAB23*	275	376	847	613	1.166959918	0.003028598	2.24538046
ENSG00000154945.7	chr17	*ANKRD40*	1033	1353	615	535	−1.047246086	0.003763125	0.483890967
ENSG00000088035.18	chr1	*ALG6*	555	393	235	223	−1.004377021	0.004432254	0.498485339
ENSG00000115540.15	chr2	*MOB4*	1198	674	426	460	−1.017715911	0.005030099	0.493897677
ENSG00000164136.17	chr4	*IL15*	231	120	44	72	−1.527845671	0.005167722	0.346794838
ENSG00000181450.18	chr1	*ZNF678*	153	160	366	292	1.09238629	0.005878984	2.132264322
ENSG00000158769.18	chr1	*F11R*	297	242	107	133	−1.126854183	0.007000778	0.457913123
ENSG00000188015.10	chr1	*S100A3*	443	344	175	205	−1.007733775	0.007855049	0.497326849
ENSG00000174600.14	chr12	*CMKLR1*	129	120	49	44	−1.392198467	0.009583138	0.380983793
ENSG00000164342.12	chr4	*TLR3*	197	212	101	72	−1.224486333	0.009602278	0.427949856
ENSG00000183778.17	chr21	*B3GALT5*	194	234	101	81	−1.222029282	0.009729242	0.428679318
ENSG00000177685.17	chr11	*CRACR2B*	297	230	129	117	−1.060061839	0.010201636	0.479611501
ENSG00000100628.12	chr14	*ASB2*	336	276	146	151	−1.005600408	0.011315502	0.498062809
ENSG00000111319.13	chr12	*SCNN1A*	337	479	182	195	−1.109036994	0.013031662	0.463603385
ENSG00000082805.20	chr12	*ERC1*	493	776	1553	1128	1.071190507	0.013705506	2.101166527
ENSG00000128284.19	chr22	*APOL3*	313	197	118	113	−1.090860846	0.013881072	0.469481155
ENSG00000007968.7	chr1	*E2F2*	220	215	113	82	−1.1348735	0.014159193	0.455374844
ENSG00000084444.14	chr12	*FAM234B*	308	347	174	149	−1.003880647	0.018168575	0.498656878
ENSG00000176406.23	chr8	*RIMS2*	108	90	196	210	1.07319732	0.018736156	2.104091319
ENSG00000237172.4	chr16	*B3GNT9*	119	165	329	267	1.074466038	0.023837031	2.105942489
ENSG00000184545.11	chr11	*DUSP8*	37	30	97	81	1.44444506	0.024706164	2.721581161
ENSG00000144401.14	chr2	*METTL21A*	366	193	143	111	−1.078869833	0.02855733	0.473399526
ENSG00000151632.17	chr10	*AKR1C2*	181	123	68	64	−1.156770825	0.030183721	0.448515322
ENSG00000163536.12	chr3	*SERPINI1*	76	68	21	26	−1.580916392	0.031445042	0.334269495
ENSG00000087301.9	chr14	*TXNDC16*	145	168	62	75	−1.172285996	0.034027509	0.443717699
ENSG00000154556.18	chr4	*SORBS2*	148	123	64	54	−1.165343942	0.035504677	0.445857953
ENSG00000239713.9	chr22	*APOBEC3G*	109	67	38	22	−1.505848746	0.035564444	0.352122972
ENSG00000134339.8	chr11	*SAA2*	105	71	26	36	−1.453948879	0.036304298	0.365020937
ENSG00000217555.12	chr16	*CKLF*	111	31	11	22	−2.00459454	0.037683108	0.249205093
ENSG00000134755.17	chr18	*DSC2*	41	36	98	90	1.320401665	0.039564399	2.497356297
ENSG00000063180.9	chr19	*CA11*	91	57	28	22	−1.516702325	0.045645702	0.349483845
ENSG00000173267.14	chr10	*SNCG*	343	175	127	117	−1.022838733	0.049724318	0.492147021
